# Effects of *Nelumbo nucifera* Gaertn. Petal Tea Extract on Hepatotoxicity and Oxidative Stress Induced by Mancozeb in Rat Model

**DOI:** 10.3390/toxics11060480

**Published:** 2023-05-25

**Authors:** Pimchanok Nuchniyom, Ketsarin Intui, Jiraporn Laoung-on, Churdsak Jaikang, Ranida Quiggins, Kornravee Photichai, Paiwan Sudwan

**Affiliations:** 1 Department of Anatomy, Faculty of Medicine, Chiang Mai University, Chiang Mai 50200, Thailand; pimchanok_nuchni@cmu.ac.th (P.N.);; 2 Department of Toxicology, Faculty of Medicine, Chiang Mai University, Chiang Mai 50200, Thailand; 3 Center of Veterinary Diagnosis and Technology Transfer, Chiang Mai University, Chiang Mai 50200, Thailand

**Keywords:** pesticides, mancozeb, toxicity, *Nelumbo nucifera*, liver, oxidative stress, glutathione

## Abstract

Mancozeb (Mz) is one of the most widely used pesticides that has been reported to cause adverse human health risks. White *Nelumbo nucifera* (*N. nucifera*) petals have therapeutic properties to prevent toxicity. Hence, this study attempted to determine the effects of *N. nucifera* extract on hepatotoxicity and oxidative stress in mancozeb-treated rats. Seventy-two male rats were divided into nine groups and designed with a control; *N. nucifera* extract was administered at the doses of 0.55, 1.1, and 2.2 mg/kg bw/day, Mz was administered at 500 mg/kg bw/day, and the co-treatment groups (*N. nucifera* and Mz) were administered 0.55, 1.1, and 2.2 mg/kg bw/day of *N. nucifera* followed by administering Mz 500 mg/kg bw/day daily for 30 days. The results showed that all doses of *N. nucifera* extract did not induce hepatic toxicity and could suppress the toxicity of mancozeb by increasing body weight gain and decreasing relative liver weight, lobular inflammation, and total injury score. The combination treatment also decreased the molecular markers of oxidative stress (2-hydroxybutyric acid, 4-hydroxynonenal, l-tyrosine, pentosidine, and N6-carboxymethyllysine). Furthermore, the reduced glutathione and oxidized glutathione contents were adjusted close to the normal level. Therefore, *N. nucifera* extract is a natural antioxidant supplement that could decrease the toxicity of mancozeb and can be safely consumed.

## 1. Introduction

No one in the human population can completely avoid exposure to pesticides. Concerns have been significantly rising about health risks over the potentially toxic effects of pesticides associated with occupational exposure through several routes. Mancozeb (Mz), which is one of the most imported fungicides in Thailand [1] is an antifungal agent that is metal-containing (manganese and zinc) and belongs to the Dithiocarbamates group. It has been widely used since the 1940s [2]. The metal ions have oxygen-transferring features to produce highly reactive hydroxyl radicals in Fenton reactions [3] or Fenton-like reactions, which decompose H_2_O_2_ into a hydroxyl radical (HO•) [4]. HO• is one of the most harmful radicals, as it is able to react with the main molecules, including proteins and lipids [5]. Additionally, previous studies have reported that it can cause toxicity in several organs [6,7,8].

The liver is the most sensitive and primary target organ regarding pesticide toxicity [9]. This organ also serves vital purposes; it is the primary site of the biotransformation process and prevents chemical toxicity through the detoxification system [10] due to its high metabolic potential (phases I and II reactions) [11]. Hence, for toxicological and pharmacological testing, hepatocytes are an obvious option [12].

In a prior study that reported the toxicity of Mz, the relative liver weight increased, while body weight gain declined. Aspartate aminotransferase (AST) and alanine aminotransferase (ALT) were altered in a concentration-dependent manner [13]. Mz also affected the histological features of the liver; 500 mg/kg of Mz induced hepatocyte degeneration, inflammation, and venous dilatation [14]. The antioxidant enzyme activity was reduced, which indicated increased lipid peroxidation (LPO) in rats exposed to Mz at doses of 250 and 500 mg/kg for 40 days [15]. Mz provokes redox homeostasis imbalance [7], and GSH content was significantly decreased after the exposure of 30 mg/kg of Mz [16]. Since the major component of Mz is metal ions that involve oxidative stress phenomena [17], advanced oxidation protein products (AOPPs) [16,18], and advanced glycation end products (AGEs) [19] are present in the reaction. Thus, these products are considered to be markers of oxidative stress.

*Nelumbo nucifera* (*N. nucifera*) is one of the most meaningful plants; its fruits and pollen have a high economic value, but its petals are leftover as agricultural waste. White *N. nucifera* petal aqueous extract has the highest total phenolic content, total tannin content, and maximal antioxidant activity, which it contains more of than red *N. nucifera* [20]. Polyphenols and flavonoids, which are commonly found in several plants, are able to scavenge ROS, thereby resulting in the inhibition of oxidative stress formation [21]. Flavonoids have anti-inflammatory properties, because they inhibit inflammatory transcription factors, reduce tissue damage and fibrosis caused by radicals [22], and regulate the cellular redox state [23]. The lotus-seed-treated group was found to lower the body weight loss of rats induced using carbon tetrachloride [24], while lotus leaf extracted with hot water was found to decrease the relative weights of retroperitoneal adipose tissues and epididymis [25]. Furthermore, the flavonoid extract from *N. nucifera* leaves reduced AST and ALT levels in the D-galactose-induced group [26]. *N. nucifera* was reported to improve histological features by reducing hepatic necroinflammation and steatosis [27]. The antioxidant properties of *N. nucifera* lotus leaves have been confirmed in their antioxidative stress by inhibiting LPO in hepatocyte cells [28]. In an in vitro study, a solution of 70% ethanol of *N. nucifera* flowers decreased malondialdehyde and maintained GSH and glutathione peroxidase to the normal level in isolated perfused rat kidneys that were induced by a Fenton mixture [29].

Due to the limited knowledge of the antioxidant potential of *N. nucifera* in relation to the complications resulting from the intake of Mz, this study aimed to investigate the potential protective effects of natural *N. nucifera* extract on mancozeb-induced hepatotoxicity and oxidative stress in male rats.

## 2. Materials and Methods

### 2.1. Ethics Statement

All protocols were approved by the Animal Ethics Committee, Faculty of Medicine, Chiangmai University (approval No. 21/2021), and according to the Guides for the Care and Use of Laboratory Animals.

### 2.2. Plant Collection, Extraction, and Phytochemical Analysis

White *N. nucifera* lotuses were collected from the Thung Yang subdistrict, Laplae district, Uttaradit Province, Thailand, in September 2019. Only complete petals were selected, washed, steamed, and dried at 60 °C. Dried petals were pulverized and stored at 4 °C in an opaque bottle before use. *N. nucifera* extract was soaked with hot distilled water at 75–80 °C for five minutes. Then, the solutions were filtered to remove insoluble particles. The extract solution was lyophilized by a Lyophilizer Lyotrap (LTE Scientific Ltd., Oldham, UK), which resulted in a 12.5% yield that was diluted before experimentation [30]. The phytochemical constituents from the extract were measured using liquid chromatography mass spectrometry (LC-MS) to investigate *N. nucifera* compositions, as reported in previous study [20].

### 2.3. Animals and Experimental Design

Seventy–two adult male Wistar rats aged 6–8 weeks, weighing 200–250 g, were purchased from Nomura Siam International Co., Ltd. (Bangkok, Thailand). Two to three rats were housed in each cage under standard environmental conditions (25 ± 2 °C temperature and 12-h light/dark cycle) and fed a standard diet and filtered water. After one week of acclimatization, the rats were divided into 9 groups (*n* = 8 per group) (none significantly differed in initial body weight among the groups at *p <* 0.05) as follows:

- Group I (control): rats in this group received 1 mL/day of distilled water.
- Group II, III, and IV (*N. nucifera* extract): rats were administered *N. nucifera* extract at doses 0.55, 1.1, and 2.2 mg/kg bw/day, which corresponds to the daily tea consumption in humans (1.1 mg/kg bw/day) [31].
- Group V (olive oil): rats were given 1 mL/day of olive oil as a vehicle group [15].
- Group VI (Mz and olive oil): the rats obtained 500 mg/kg bw/day of Mz; the dose of Mz was chosen due to its ability to induce liver toxicity [32].
- Group VII, VIII, and IX *(N. nucifera* extract and Mz): rats were treated with *N. nucifera* extract 0.55, 1.1, 2.2 mg/kg bw/day followed by 500 mg/kg bw/day of Mz.

All groups were orally treated by gavage needle daily for 30 days.

### 2.4. Body Weight, Liver Weight, and Relative Liver Weight Analysis

The body weight data was recorded every 3 days from arrival until they were sacrificed. At the end of the experiment period, the animals were anesthetized using Isoflurane and sacrificed. Their abdominal cavities were opened to carefully harvest the livers, then cleaned and weighed. The relative liver weights were calculated by dividing each liver weight by their body weight.

### 2.5. Determination of Liver Function

Blood samples of 3 mL were taken from the left ventricle and stored in EDTA blood tube. The serums AST and ALT were measured using an Automated Clinical Chemistry Analyzer BX-3010 (Sysmex Asia Pacific Pte Ltd., Singapore) at Hematology and Biochemistry Laboratory of Small Animals Hospital, Faculty of Veterinary Medicine, Chiang Mai University.

### 2.6. Liver Histopathological Evaluation

After being sacrificed, the livers were quickly dissected and fixed in 10% formaldehyde. The tissues were processed according to the paraffin technique, wherein they were dehydrated through ascending grades of ethanol, cleaned in xylene, embedded in paraffin wax, segmented at 3 µm by rotary microtome, and stained with hematoxylin and eosin (H and E). For microscopic examination, photomicrographs were taken at 400× using an Olympus CH-BI45-2 digital microscope camera (Olympus, Tokyo, Japan), and image capture was taken with a UCMOSO3100KPA Toupcam microscope digital camera (ToupTek Company, Zhejiang, China) at Research Unit I, Faculty of Medicine, Chiang Mai University. The blind slides also investigated the degree of hepatic injury under a light microscope; grading and scoring (<5% = 0, 5−33% = 1, 33–60% = 2, >60% = 3) was documented according to The Nonalcoholic Steatohepatitis Clinical Research Network (The NASH CRN) [33] by a veterinary pathologist.

### 2.7. Oxidative Stress Molecular Markers and Glutathione Levels Measurement

**Figure 1:**
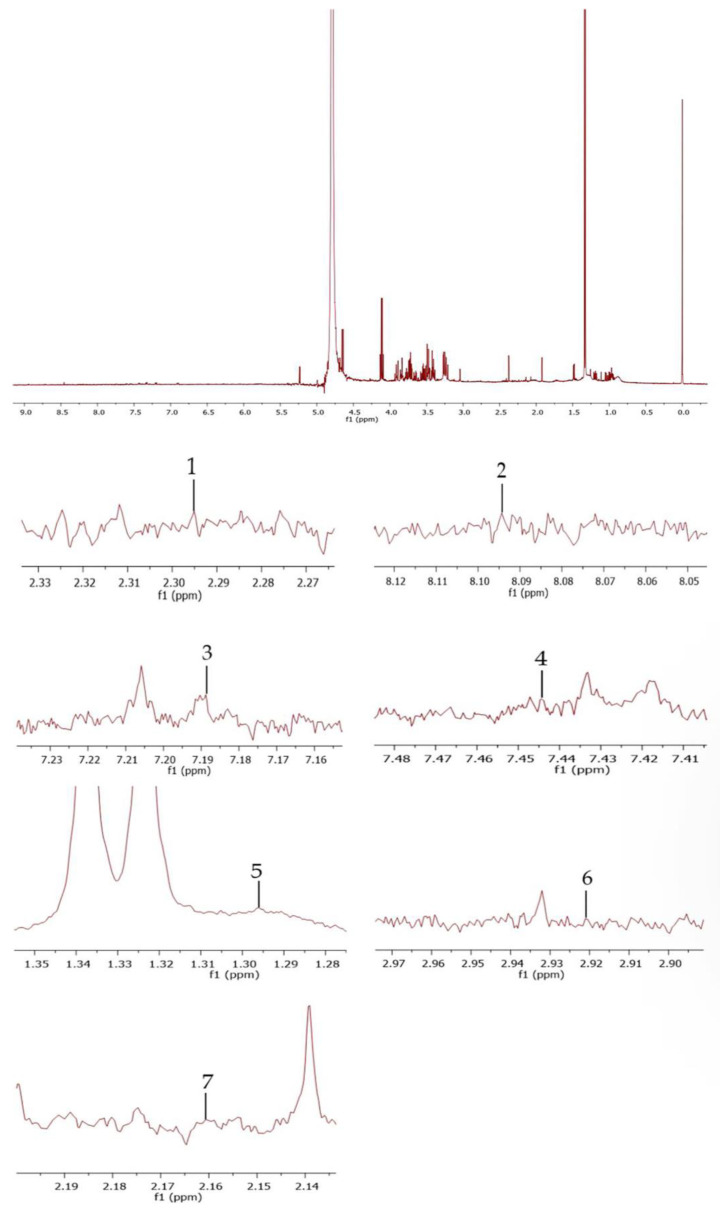
The 1H-qNMR spectra from blood serum show peak identification: peak 1—2-hydroxybutyric acid; peak 2—4-HNE; peak 3—l-tyrosine; peak 4—pentosidine; peak 5—N6-carboxymethyllysine; peak 6—reduced glutathione; and peak 7—oxidized glutathione.

The markers of oxidative stress, including 2-hydroxybutyric acid, 4-hydroxynonenal (4-HNE), l-tyrosine, pentosidine, and N6-carboxymethyllysine, as well as the reduced and oxidized glutathione contents from blood serum, were conducted using a Bruker Advance Neo 500 MHz (Bruker Daltonics, Billerica, MA, USA) nuclear magnetic resonance spectrometer (NMR) using deuterium oxide (D_2_O) as the test solvent and trimethylsilylpropanoic acid (TSP) as the standard internal substance. A 1H-qNMR examination was performed on a 500 MHz spectrometer. The quantitative resonance peaks of each aforementioned molecular marker were at δ2.30 ppm, δ8.09 ppm, δ 7.19 ppm, δ7.44 ppm, δ1.30 ppm, δ2.92 ppm, and δ2.16 ppm, respectively (Figure 1). The data was processed using MestReNova 11.0.4 software (Mestrelab Research S.L., Santiago de Compostela, Spain) to specify peaks, analyze the spectra, and calculate sample concentrations [34].

### 2.8. Statistical Analysis

The data were presented as descriptive statistic mean ± standard error (SE). The normal distribution was examined by the Kolmogorov–Smirnov test. Mean values were analyzed using one-way ANOVA following Duncan’s multiple-range test for normality. The mean values of the other parameters, which are non-normality, were measured using the Kruskal–Wallis test, followed by Mann–Whitney U tests, which were performed to analyze the differences between groups. SPSS 22 statistical software (Chicago, IL, USA) was applied for all statistical analyses. A difference of *p* < 0.05 was considered statistically significant.

## 3. Results

### 3.1. Effects of N. nucifera Extract on Body Weight

The mean values of body weight gain, liver weight, and relative liver weight are presented in Table 1 [Group I: = Control; Group II: *N. nucifera* (0.55 mg*/*kg bw*/*day); Group III: *N. nucifera* (1.1 mg/kg bw/day); Group IV*: N. nucifera* (2.2 mg*/*kg bw*/*day); Group V: olive oil; Group VI: Mz (500 mg/kg bw/day); Group VII: *N. nucifera* (0.55 mg/kg bw/day) and Mz (500 mg/kg bw/day); Group VIII: *N. nucifera* (1.1 mg/kg bw/day) and Mz (500 mg/kg bw/day); Group IX: *N. nucifera* (2.2 mg/kg bw/day) and Mz (500 mg/kg bw/day)]. The body weight gain results showed that groups II, III, IV, and V increased significantly *(p* < 0.05) when compared with group VI. In contrast, there was a significant (*p* < 0.05) decrease in groups VI, VII, and IX when compared to group I. There were no statistically significant differences in liver weight between groups (*p* < 0.05). Additionally, the relative liver weight was found to be reduced with statistical difference (*p* < 0.05) in groups II, III, IV, and V when compared to group VI. Group VI, VII, and VIII were significantly higher (*p* < 0.05) than group I.

### 3.2. Effects of N. nucifera Extract on Liver Function Parameters

In this experiment, the concentrations of hepatic enzymes (AST and ALT) were not significantly different in all groups at *p* < 0.05, as shown in Table 2.

### 3.3. Effects of N. nucifera Extract on Histopathological Features and Total Score Injury of Livers

**Figure 2:**
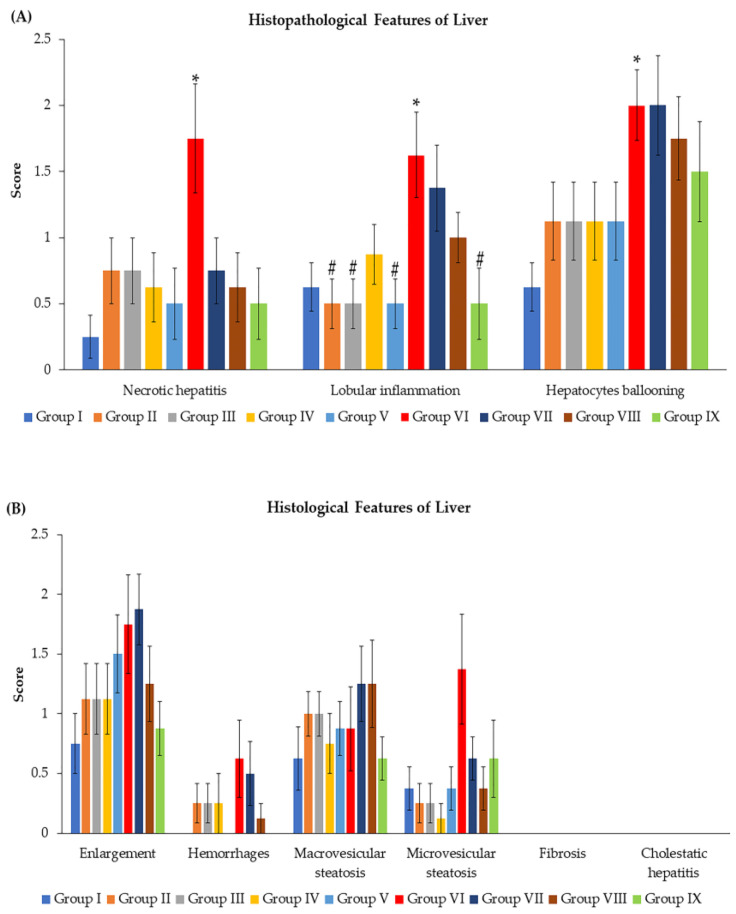
Effects of *N. nucifera* extract on histopathological features of livers. (**A**) histopathological features of livers; necrotic hepatitis, lobular inflammation, and hepatocellular ballooning expressed as mean score ± SE. (**B**) histopathological features of livers; enlargement, hemorrhage, macro- and micro-vesicular steatosis, fibrosis, and cholestatic hepatitis as mean score ± SE. The results are shown as mean ± SE of *n* = *8*. * *p* < 0.05 vs. Group I; ^#^ *p* < 0.05 vs. Group VI.

The mean values of necrotic hepatitis, lobular inflammation, and hepatocellular ballooning scores are shown in Figure 2A; all the above parameters significantly increased in group VI (*p* < 0.05) when compared with group I. The data for lobular inflammation scores revealed that groups II, III, V, and IX were considerably lower (*p* < 0.05) than group VI. There were no significant differences between all groups in enlargement, hemorrhage, macro- and micro-vesicular steatosis, fibrosis, and cholestatic hepatitis scores at *p* < 0.05, as demonstrated in Figure 2B.

**Figure 3:**
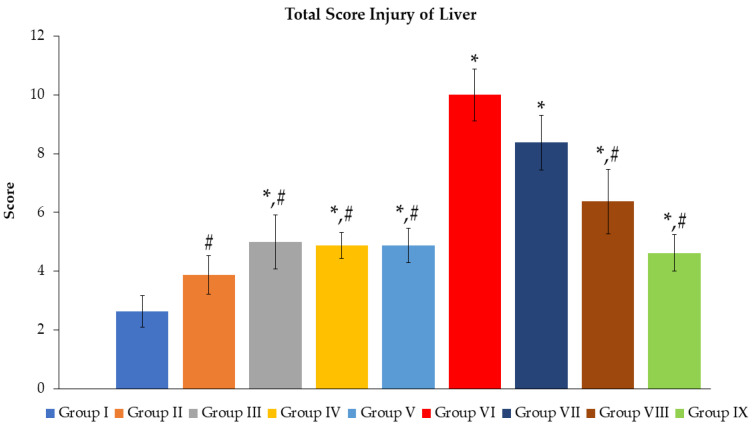
Effects of *N. nucifera* extract on total score injury of liver. The findings are presented as mean ± SE of *n = 8*. * *p* < 0.05 vs. Group I; ^#^ *p* < 0.05 vs. Group VI.

**Figure 4:**
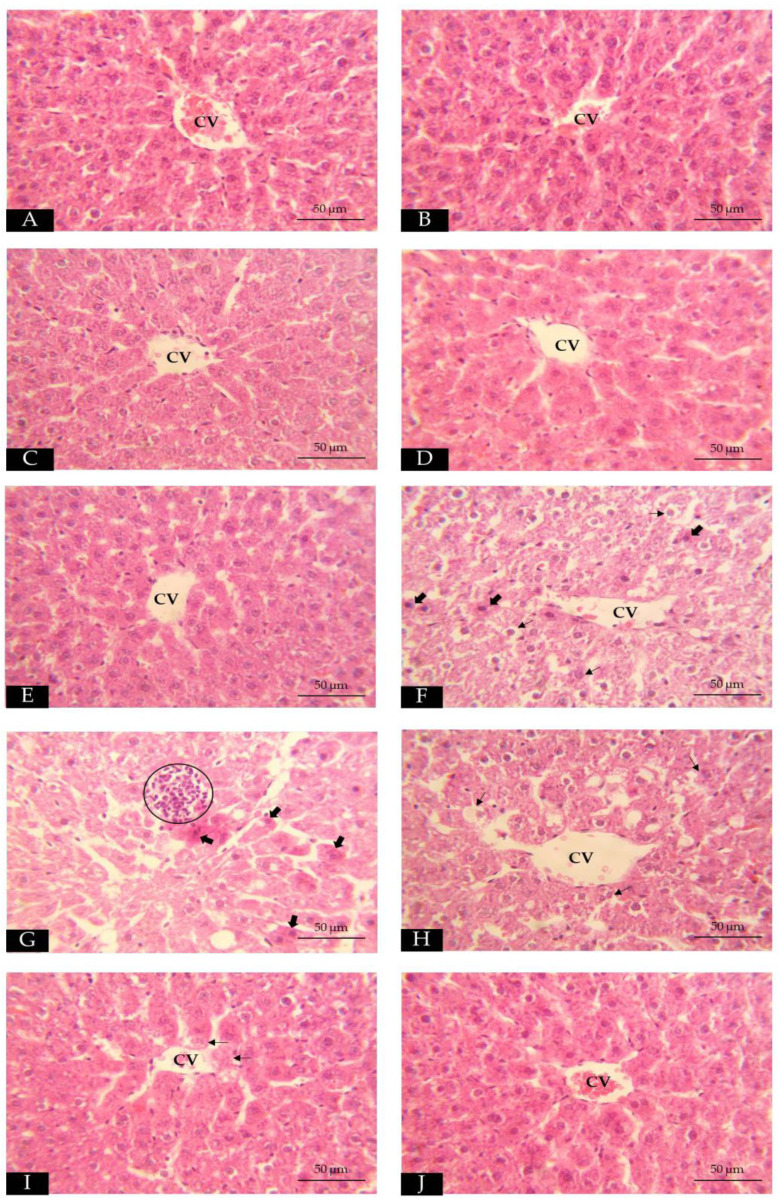
Photograph of histological changes of liver using hematoxylin and eosin (**E**,**H**) staining; 40× scale bar 50 µm. (**A**): group I; the central vein (CV) of the liver lobule is surrounded by hepatocytes with a regular hepatic architecture. (**B**): group II, (**C**): group III, (**D**): group IV, and (**E**): group V presented normal histology of central veins and hepatocytes. (**F**,**G**): group VI; the liver tissues of this group showed the disorganization of the hepatic parenchymal, necrotic hepatitis (filled black arrow), hepatocellular ballooning (black arrow), and macrophage aggregation (circle). (**H**): group VII; the hepatocytes displayed hepatocellular ballooning. (**I**): group VIII; the livers had mild hepatocellular ballooning. (**J**): group IX; typical hepatocytes almost as group I.

The mean of total score injury (Figure 3) revealed that, although all groups were significantly increased (*p* < 0.05) when compared to group I, Groups III–V were significantly lower than group VI (*p* < 0.05). The oral administration of Mz led to remarkably elevated scores (*p* < 0.05) in groups VI–IX in comparison with group I; however, this tended to reduce from group VI with statistical differences at *p* < 0.05 in groups VIII and IX. Likewise, we carried out histopathological observations, as demonstrated in Figure 4. Group I showed the typical hepatocytes radiating out from a central vein (Figure 4A). None of the abnormal structures of hepatocytes and central veins in groups II, III, IV (Figure 4B–D), and group V (Figure 4E) appeared. Mz caused the disorganization of the hepatic parenchymal and altered the liver cell morphologies; necrotic hepatitis, hepatocellular ballooning, and lobular inflammation were found in group VI (Figure 4F–G). Group VII (Figure 4H) and group VIII (Figure 4I) featured mild hepatocellular ballooning. Group IX illustrated the normal structure of hepatocytes and was nearly identical to group I (Figure 4J).

### 3.4. Effects of N. nucifera Extract on Oxidative Stress Molecular Markers

**Figure 5:**
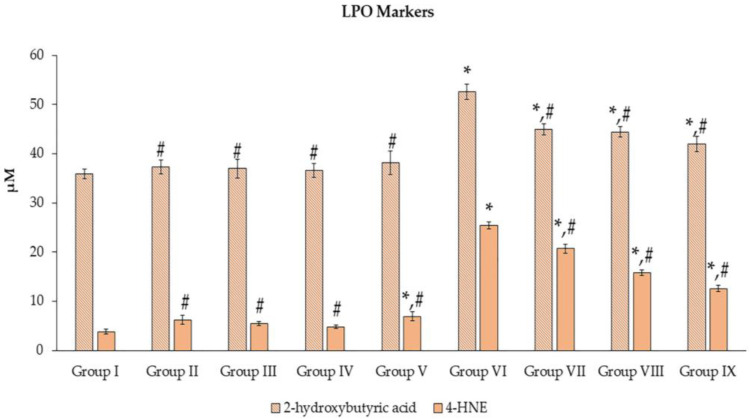
Effects of *N. nucifera* extract on LPO molecular markers. The values are shown as mean ± SE of *n* = *8*. * p < 0.05 vs. Group I; ^#^ *p* < 0.05 vs. Group VI.

LPO reaction was analyzed by measuring 2-hydroxybutyric acid and 4-HNE, as shown in Figure 5. The induction of Mz brought about a significant increase (*p* < 0.05) in all experiment groups when compared to group I. However, when the extract was combined with Mz (groups VII–IX), there was a significant attenuation (*p* < 0.05) of both LPO indicators compared with group VI. In groups II–IV, the markers were significantly lower (*p* < 0.05) than in group VI. Additionally, the 4-HNE parameter in group V was slightly higher (*p* < 0.05) than in group I.

**Figure 6:**
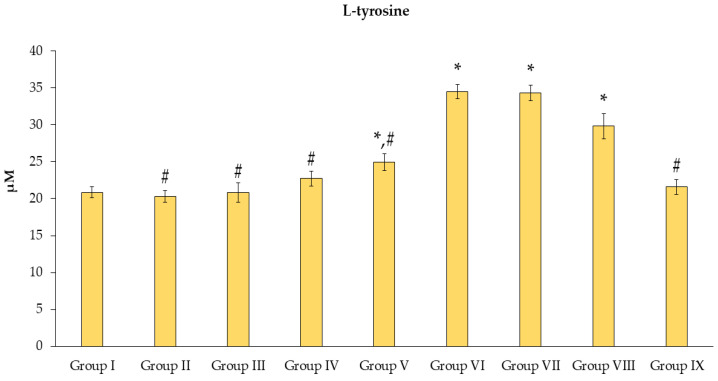
Effects of *N. nucifera* extract on AOPP molecular markers. The data are reported as mean ± SE of *n = 8*. * *p* < 0.05 vs. Group I; ^#^ *p* < 0.05 vs. Group VI.

The markers of AOPPs and l-tyrosine (Figure 6) were investigated, and the data revealed that the Mz caused significantly elevated concentrations (*p* < 0.05) when compared with group I. All groups had fewer concentrations of l-tyrosine than group VI (*p* < 0.05), except for groups VII and VIII, when compared to group VI. The differences in l-tyrosine levels between groups VII, VIII, and VI were not significant (*p* < 0.05). Group V exhibited a mild increase (*p* < 0.05) when compared to group I. Interestingly, group IX showed a considerable alleviation when compared to group VI at *p* < 0.05.

**Figure 7:**
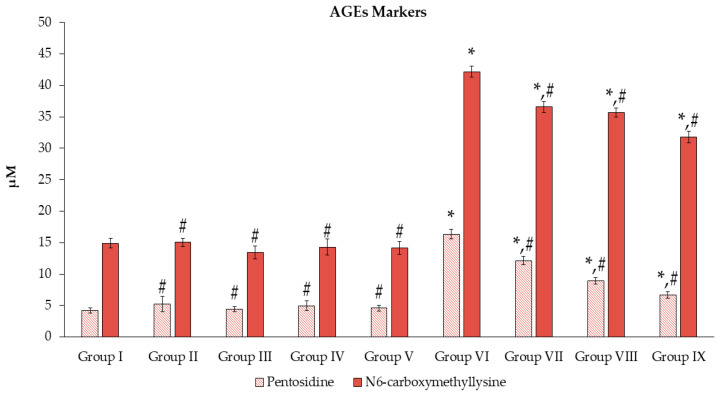
Effects of *N. nucifera* extract on AGE molecular markers. The results are presented as mean ± SE of *n* = *8*. * *p* < 0.05 vs. Group I; ^#^ *p* < 0.05 vs. Group VI.

Pentosidine and N6-carboxymethyllysine refer to AGE phenomenon; the mean values of these indexes are illustrated in Figure 7. Groups III–V exhibited significantly lower levels of these markers when compared to group VI (*p* < 0.05). Exposure to Mz led to significantly higher levels (*p* < 0.05) of both markers in groups VI–IX when compared with group I. Meanwhile, treatment with the different doses of the extract significantly declined (*p* < 0.05) the levels of these markers when compared to group VI.

### 3.5. Effects of N. nucifera Extract on Glutathione Levels

**Figure 8:**
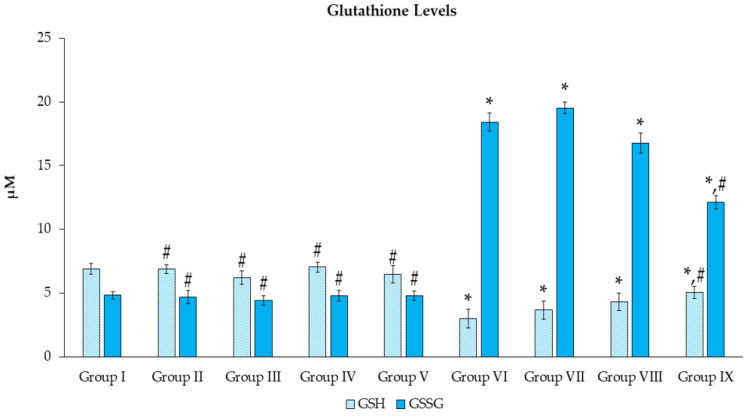
Effects of *N. nucifera* extract on glutathione levels. The findings are expressed as mean ± SE of *n = 8*. * *p* < 0.05 vs. Group I; ^#^ *p* < 0.05 vs. Group VI.

The reduced and oxidized glutathione concentrations were measured (Figure 8), and the results showed no differences with statistical significance in groups II–V when compared to group I. The administration of Mz caused the reduced glutathione contents to significantly decline (*p* < 0.05) in groups VI–IX, while it led to a remarkable increase (*p* < 0.05) in GSSG concentrations when compared to group I. Noticeably, reduced glutathione levels were elevated in group IX (*p* < 0.05), along with a reduced rate of oxidized glutathione, when compared with group VI (*p* < 0.05).

## 4. Discussion

Mz exposure is an ongoing problem that can bring about serious health risks in the long run. For this reason, natural products have raised great attention to attenuate Mz toxicity due to their antioxidant properties. In the current study, we examined the effects of *N. nucifera* on the livers of Mz-treated rats in order to better understand their hepatoprotective capacity by investigating weight, liver function biomarkers, histopathological changes, oxidative stress molecular markers, and glutathione contents.

Body weight gain [35] alteration is regarded as a sensitive indicator of toxic-induced pathology. Generally, relative organ weights were estimated as a critical prerequisite to compare the effect of xenobiotics on individual organs between the treated and untreated groups [36]. Our result reported that the *N. nucifera* extract did not affect those markers; the findings agree well with prior research that reported the safe use of *N. nucifera* fruit ethanol extract on body weight in rat models [37]. Our study found that Mz exposure was toxicologically relevant to body weight gain and relative liver weight. As illustrated in previous research, Mz inhibited the percentage of body weight gain in rats [38]. To support these findings, fungicides, such as Mz, are endocrine-disrupting chemicals that alter growth rates via a hormone function disturbance mechanism [39]. These results were similar to another study that revealed an increase in the relative liver weight after obtaining 500 mg/kg bw/day of Mz [32]. The low body weight gain was proportional to the loss of final body weight associated with increasing relative organ weights [40]. Nonetheless, N. nucifera extract combined with Mz tended to improve those parameters to the control level.

The rates of AST and ALT were quantified, as these enzymes are an indicator of liver damage; as a result of injury, the enzymes increased and migrated into the circulation [41]. The extract did not alter these liver enzyme levels, which is consistent with a previous report where *N. nucifera* fruits administered in amounts of 50 and 100 mg/kg bw/day did not change the AST or ALT contents [37]. In the present study, Mz amounts at 500 mg/kg bw/day did not produce any significant difference with group I; in contrast to earlier research by Yahia et al., the levels of AST and ALT increased after Mz administration at doses of 800 and 1200 mg/kg exposed in rats for 8 weeks [42], which may have been due to the lower concentrations of Mz and the short experiment duration. Although the biochemical analyses remained unchanged, the liver’s histology had altered similarly to the prior findings; the toxicity of *Kaempferia parviflora* extract caused the variations in the liver histology of rats, although there were no changes in the levels of AST and ALT [43].

The current study of cell morphology levels found necrotic hepatitis, lobular inflammation, and hepatocellular ballooning after obtaining Mz, which were in accordance with earlier research that illustrated the focal necrosis of hepatocytes in Mz-treated rats [14]. The hepatotoxicity of Mz on histological features such as inflammatory infiltration and balloonization has also been reported [44]. The total score injury was highest in group VI only; this result was supported by prior evidence on another ROS-generating substance [24]. The detrimental histological effects induced by Mz may be related to ROS accommodated during oxidative stress reaction; the balance between ROS and their scavengers is interrupted, and the overwhelming product can damage key biomolecules, which then causes apoptosis, inflammation, or necrosis [45]. Damage to the cytoskeleton or the membrane can induce hepatocellular ballooning, which results in the loss of cell structure and allows fluid into the cell [46]. In this animal model experiment, the other parameters were not obviously different; this is in contrast with the previous results from an in vitro study, which demonstrated fatty-acid-induced steatosis in hepatocarcinoma cell lines [47]. Since liver fibrosis is associated with chronic liver injury [48], and cholestatic hepatitis commonly manifests 2 to 12 weeks after exposure [49], these histologic features were not found in our study because of the short period. Our data revealed that the liver cell morphology was unaffected by the *N. nucifera* extract. Additionally, it had a hepatoprotective ability against Mz toxicity by reducing the hepatocyte inflammatory and total injury score compared to group VI. This might have occurred via a ROS scavenging mechanism of antioxidant substances, including phenolic compounds [50], which are a prominent constituent in this extract.

NMR has become a “gold standard” for metabolomic studies in just three decades, especially in pharmacological and medical fields [51]. Our research applied this device to confirm the existence of oxidative stress reactions: LPO, AOPPs, and AGEs refer to adverse impacts on the primary biomolecules of lipids, proteins, and carbohydrates, respectively. LPO was analyzed by measuring the 2-hydroxybutyric acid and 4-HNE. 2-hydroxybutyric acid that emerged as a byproduct of raising hepatic glutathione stress and increasing the ratio of the reduced and oxidized forms of nicotinamide adenine dinucleotide (NADH/NAD and ratio) from elevated lipid oxidation [52]. The 4-HNE is also a secondary product generated during polyunsaturated fatty acid peroxidation [53]. The findings indicated no significant difference between the lotus extract groups (groups II, III, and IV) and the control group (group I). It implied that the *N. nucifera* extract did not influence the oxidative degradation of the lipids. At the same time, exposure to Mz caused lipid oxidation to be exacerbated; previous studies support our results [15,54]. The risk of LPO mainly belongs to the formation of ROS. These radicals are impacted structures, and the dynamics of the phospholipid bilayer membrane [55] was a possible mechanism.

In a recent study, we evaluated l-tyrosine content as an AOPP marker; this amino acid residue may oxidize, thereby changing the structure of proteins and causing them to cross-link, aggregate, or fragment under oxidative stress conditions such as AOPP reaction [56]. Compared to group I, three dosages of *N. nucifera* extract did not contribute to any differences. Conversely, Mz brought about increased l-tyrosine levels when compared with group I, which is consistent with the prior insecticide research that 25 mg/kg of cypermethrin treated in rats resulted in considerably higher AOPP levels in liver and kidney [57]. It has been reported that proteins are major targets for oxidation processes because of their large scale and rapid interactions with oxidants [58]. In addition, tyrosine contains the phenolic side-chain, which readily oxidizes and then combines to yield dityrosine cross-links in protein, which is a biomarker of oxidative stress [59].

AGEs are categorized as fluorescent AGEs (pentosidine) and nonfluorescent AGEs (N6-carboxymethyllysine), which are related to the glycation reaction of glucose or other saccharide derivatives with lipids or protein during the Maillard reaction [60]. Similar to the results of the LPO and AOPPs, three different doses of *N. nucifera* extract did not produce any alteration when compared to group I. Meanwhile, the administration of Mz led to remarkably increased markers of glycoxidation. The results were corroborated by the previous evidence; AGEs were formed as a result of free radicals and metal-catalyzed oxidation [61]. The underlying mechanisms of this conclusion may be due to glyoxal, which is an AGE precursor that originated in metal ions catalyzed by the autoxidation of glucose, which is known as the so-called Wolff pathway [62].

The *N. nucifera* extract could prevent Mz-induced oxidative stress by inhibiting LPO, AOPPs, and AGEs. The present study was confirmed by previous results, which demonstrated the antioxidant properties of white *N. nucifera* extract that significantly decreased both LPO and AOPP contents in an in vitro model [20]. Other data from in vitro research suggested that *N. nucifera* leaf extract treatments inhibited AGE generation on rat using lens aldose reductase [63]. White *N. nucifera* extract is rich in antioxidants, particularly phenolics and total tannins, which support these capacities. These compounds play a critical role in defending against cell damage caused by oxidative stress through scavenging ROS/singlet oxygen and metal chelating, especially for the most hazardous oxygen free radicals, HO•, which is a product of the reaction between transition metal ions and H_2_O_2_ (Fenton reaction), including guarding against the Fenton-like reactions that trigger the release of alkoxyl radicals (LO•) from the degradation of lipid hydroperoxides [64].

GSH is the essential hydrophilic antioxidant shielding cells against external and internal harmful substances, including ROS, and serves a purpose in regulating the balance of redox status [65]. Our findings reported that groups II–IV were not significantly different compared to group I in all parameters. GSH decreased considerably compared to group VI in groups VII–IX. On the other hand, those groups were higher than group I in GSSG levels. The earlier evidence in another pesticide supported our data; in an in vitro experiment, exposure to methomyl significantly decreased GSH but elevated GSSG levels [66]. We have observed that *N. nucifera* extract was found to adjust the GSH and GSSG close to the normal range in groups VII–IX when compared to Mz-treated alone. As Mz consists of transition metal ions that combine with peroxides to influence ROS accumulation, antioxidants such as glutathione are crucial in diminishing radicals [67]. In oxidative stress conditions, not only is an excessive amount of reactive oxygen species (ROS) unable to be completely eliminated, leading to cell damage, but there is also increased demand for glutathione. This prompts the conversion of GSSG to GSH through the glutathione reductase pathway [68]. Thus, GSH is reduced in parallel with increasing GSSG. The lotus extract possesses an antioxidant constituent that has been reported for rebalancing ROS and its scavengers to the regular proposition and promoting the expression of phase II detoxifying enzymes by triggering the nuclear translocation of nuclear factor erythroid 2-related factor 2 (Nrf2) [69], which supports the current results.

## 5. Conclusions

In conclusion, the current study indicated the effects of *N. nucifera* extract at different doses on hepatotoxicity and oxidative stress caused by Mz. The extract was safely used and suppresses Mz toxicity on body weight, histological changes, oxidative stress molecular markers, and glutathione contents, probably via ROS scavenging and metal chelating, as well as by stimulating phase II detoxifying enzyme expression processes. However, the dosage effects, duration, and underlying mechanisms still require further study. *N. nucifera*, a local plant, has a great probability of developing into a valuable supplement of natural antioxidants against Mz toxicity. They have high pharmaceutical properties, as well as their affordability for people who have high-risk exposure to Mz, especially cultivators or individuals who desire to use it as an additional natural foodstuff.

## Figures and Tables

**Table 1:** Effects of *N. nucifera* extract on body weight. The values are expressed as mean ± SE.

Group	Body Weight Gain (%)	Liver Weight (g)	Relative Liver Weight (%)
Group I	79.46 ± 6.16	14.38 ± 0.80	3.44 ± 0.14
Group II	75.53 ± 3.55 ^#^	14.67 ± 0.71	3.57 ± 0.07 ^#^
Group III	72.73 ± 1.40 ^#^	13.82 ± 0.51	3.43 ± 0.10 ^#^
Group IV	76.62 ± 2.41^#^	14.14 ± 0.42	3.51 ± 0.08 ^#^
Group V	87.81 ± 8.75 ^#^	15.16 ± 0.75	3.45 ± 0.15 ^#^
Group VI	56.22 ± 3.39 ^#^	14.87 ± 0.44	4.01 ± 0.10 ^#^
Group VII	55.25 ± 9.62 *	15.21 ± 1.24	4.13 ± 0.12 *
Group VIII	64.60 ± 2.90	15.05 ± 0.68	3.85 ± 0.11 *
Group IX	56.82 ± 3.80 *	12.72 ± 0.48	3.75 ± 0.14

Values are presented as mean ± SE of *n* = *8*. * *p* < 0.05 vs. Group I; *^#^ p* < 0.05 vs. Group VI.

**Table 2:** Effects of *N. nucifera* extract on liver function. All values are reported as mean ± SE.

Group (*n* = *8*)	AST (U/L)	ALT (U/L)
Group I	171.37 ± 15.25	63.00 ± 10.14
Group II	220.12 ± 42.06	71.50 ± 10.93
Group III	151.87 ± 14.70	52.00 ± 5.16
Group IV	226.25 ± 64.16	54.62 ± 8.98
Group V	184.37 ± 11.25	53.12 ± 6.12
Group VI	175.37 ± 19.12	70.37 ± 11.77
Group VII	159.50 ± 23.87	46.25 ± 8.03
Group VIII	194.50 ± 38.46	68.87 ± 12.14
Group IX	161.25 ± 19.44	58.25 ± 9.90

## Data Availability

All data have been included in the manuscript.
